# Target capture sequencing for the first Nigerian genotype I ASFV genome

**DOI:** 10.1099/mgen.0.001069

**Published:** 2023-07-25

**Authors:** Adeniyi C. Adeola, Pam D. Luka, Xiang-Xiang Jiang, Zheng-Fei Cai, Olufunke O. Oluwole, Xian Shi, Bukola M. Oladele, Temilola O. Olorungbounmi, Bamidele Boladuro, Oladipo Omotosho, Victor M. O. Okoro, Philip M. Dawuda, Sunday C. Olaogun, Oscar J. Sanke, Hai-Bing Xie, Richard P. Bishop, Jianlin Han, Jianbo Li, Ya-Ping Zhang, Min-Sheng Peng

**Affiliations:** ^1^​ State Key Laboratory of Genetic Resources and Evolution and Yunnan Laboratory of Molecular Biology of Domestic Animals, Kunming Institute of Zoology, Chinese Academy of Sciences, Kunming, PR China; ^2^​ Sino‐Africa Joint Research Center, Chinese Academy of Sciences, Kunming, PR China; ^3^​ Centre for Biotechnology Research, Bayero University, Kano, Nigeria; ^4^​ National Veterinary Research Institute, Vom, Nigeria; ^5^​ College of Life Sciences, Anhui Normal University, Wuhu, PR China; ^6^​ State Key Laboratory for Conservation and Utilization of Bio-resources in Yunnan, Yunnan University, Kunming, PR China; ^7^​ Institute of Agricultural Research and Training, Obafemi Awolowo University, Ibadan, Nigeria; ^8^​ Kunming College of Life Science, University of Chinese Academy of Sciences, Kunming, PR China; ^9^​ Department of Veterinary Medicine, University of Ibadan, Ibadan, Nigeria; ^10^​ Department of Animal Science and Technology, School of Agriculture and Agricultural Technology, Federal University of Technology, Owerri, Nigeria; ^11^​ Department of Veterinary Surgery and Theriogenology, College of Veterinary Medicine, University of Agriculture Makurdi, Makurdi, Nigeria; ^12^​ Taraba State Ministry of Agriculture and Natural Resources, Jalingo, Nigeria; ^13^​ International Livestock Research Institute (ILRI), Nairobi, Kenya; ^14^​ CAAS-ILRI Joint Laboratory on Livestock and Forage Genetic Resources, Institute of Animal Science Chinese Academy of Agricultural Sciences(CAAS), Beijing, PR China

**Keywords:** African swine fever virus (ASFV), genome, genotype I, Nigeria, phylogeny, target capture sequencing

## Abstract

African swine fever (ASF) is a contagious viral disease that affects domestic pigs and wild boars, causing significant economic losses globally. After the first Nigerian outbreak in 1997, there have been frequent reports of ASF in pig-producing regions in the country. To facilitate control, it is important to understand the genotype and phylogenetic relationship of ASF viruses (ASFVs). Recent genetic analysis of Nigerian ASFV isolates has revealed the presence of both genotypes I and II; this is based on analysis of a few selected genes. Phylogenetic analysis of ASFV whole genomes highlights virus origins and evolution in greater depth. However, there is currently no information on the ASFV genome from Nigerian isolates. Two ASFV-positive samples were detected during a random survey of 150 Nigerian indigenous pig samples collected in 2016. We assembled near-complete genomes of the two ASFV-positive samples using in-solution hybrid capture sequencing. The genome-wide phylogenetic tree assigned these two genomes into *p72* genotype I, particularly close to the virulent Benin 97/1 strain. The two ASFVs share 99.94 and 99.92 % genomic sequence identity to Benin97/1. This provides insight into the origin and relationship of ASFV strains from Nigeria and Italy. The study reports for the first time the determination of near-complete genomes of ASFV using in-solution hybrid capture sequencing, which represents an important advance in understanding the global evolutionary landscape of ASFVs.

## Data Summary

The published complete genomic information of ASFV are listed in the Table S1 (available in the online version of this article). We deposited the raw sequencing data at the National Center for Biotechnology Information (NCBI) with BioProject accession no. PRJNA844513 (publicly accessible at https://www.ncbi.nlm.nih.gov/bioproject/?term=PRJNA844513). These data have also been deposited at the Genome Sequence Archive (GSA) of the National Genomic Data Center (NGDC) under the project accession no. PRJCA014704 (publicly accessible at https://ngdc.cncb.ac.cn/search/?dbId=&q=PRJCA014704). The assembled genomes are available in the GenBase database of the NGDC with the accession nos C_AA008385.1 (https://ngdc.cncb.ac.cn/genbase/search/gb/C_AA008385.1) and C_AA008386.1 (https://ngdc.cncb.ac.cn/genbase/search/gb/C_AA008386.1) for ASFV Nigeria_MS001 and ASFV Nigeria_MS002, respectively.

**Impact Statement**

African swine fever (ASF) is a viral disease in domestic pigs and wild boars that results in mortality of almost all infected animals. The disease poses a huge threat to the pig industry worldwide, and no vaccine is available. To facilitate control, it is important to understand the genotype and phylogenetic relationship of ASF viruses’ (ASFVs’) whole genomes. However, there is currently no information on ASFV genomes from Nigerian isolates. This study assembled near-complete genomes of two ASFV-positive samples using in-solution hybrid capture sequencing. The genome-wide phylogenetic tree assigned these two genomes into *p72* genotype I, particularly close to the virulent Benin 97/1 strain. This provides insight into the origin and relationship of ASFV strains from Nigeria and Italy. The study reports for the first time the determination of complete genomes of ASFV using in-solution hybrid capture sequencing, which represents an important advance in understanding the global evolutionary landscape of ASFVs.

## Introduction

African swine fever (ASF) is a highly contagious disease of domestic pigs and their evolutionary precursor wild boars (*Sus scrofa*) caused by African swine fever virus (ASFV) [1, 2]. The infected pigs usually have different degrees of haemorrhagic fever, which leads to the death of pigs in severe cases, particularly when previously naïve domestic pig populations are infected [3]. This has resulted in huge losses in global porcine production and the economies of the countries involved. ASFV is the only member of the genus *Asfivirus* within the family *Asfarviridae*, and the only DNA virus known to be transmitted by arthropods, specifically *Ornithodoros* soft ticks [4]. The ASFV genome is double-stranded linear DNA between 170–194 kb in size and contains 150–198 open reading frames (ORFs) [5]. The first report of ASF was in Kenya over 100 years ago, after which the disease spread into Nigeria, probably from South Africa in 1997 [6, 7]. ASF is now endemic in Nigeria, with multiple outbreaks occurring in pig farming areas [8]. Genetic studies categorized the Nigerian ASFV isolates into genotypes I and II based on partial sequencing of the C-terminal region of *B646L* gene [9]. Phylogenetic analysis of ASFV can provide important information on virus origin and evolution, but there are some limitations in single gene-based phylogenetic reconstructions, which provide poor clarity about the origins and evolutionary relationships of some strains. The phylogeny of whole genomes can provide the clonal ancestry at higher resolution and shed light on the distribution of recombination and the associated mutation rate across the entire genome [10–12]. In-solution hybrid capture sequencing is a generally employed strategy in both clinical and research settings, as it substantially reduced sequencing and computational resources as compared to metagenome sequencing, allowing it to be a more cost-effective strategy that can be more readily implemented in ASFV genome sequencing [13, 14]. Unfortunately, ASFV genomic data are scarce in West Africa, especially in Nigeria, where p72 genotype II strains have recently emerged in addition to genotype I [15].

## Theory and implementation

### Sampling

In this study, we collected indigenous pig ear tips samples from three states in Nigeria, Oyo, Benue and Taraba, in 2016. Total DNA was extracted from all samples using the standard phenol/chloroform method [16]. Briefly, it was incubated at 56 °C with 200 µg ml^−1^ proteinase K and 1 % SDS (Sodium dodecyl sulfate) until complete digestion, and then extracted twice with phenol/chloroform. Finally, the DNA was washed with 70 % ethanol and resuspended in 50 µl TE buffer (Tris-EDTA buffer solution, pH=8.0). ASFV-positive samples were detected via PCR [8, 9, 17] and two samples (Nigeria_MS001 and Nigeria_MS002) with positive PCR signals for the ASFV *p72* gene were further subjected to next-generation sequencing.

### Next-generation sequencing

DNA libraries were constructed to have insert sizes of 350 bp, according to the manufacturer’s instructions (Illumina, USA). Sequencing was performed to generate 150 bp paired-end reads on the NovaSeq 6000 platform (Illumina, USA) according to the manufacturer’s protocol to generate 17.5 and 16.6 Gb of data for Nigeria_MS001 and Nigeria_MS002, respectively. We removed adaptor sequences with Trimmomatic v0.36 [18]. Reads with a length <30 bp were excluded. BWA-MEM [19] was used to map the reads to the host genomes (*Sus_scrofa 11.1* and *DNSE* [20]) with default parameters. Unmapped reads were extracted using SAMTOOLS v1.9 [21] with the parameter -f 4. A total of 992 906 reads from sample Nigeria_MS001 and 1 318 961 reads from sample Nigeria_MS002 were kept for mapping against the ASFV genome Benin 97/1 strain (NC_044956.1) [22] after removing the host genome reads. Duplicates were marked using the MarkDuplicates module of GATK v4.1.3.0 [23] with default parameters. Duplicate reads were removed by SAMTOOLS v1.9 [21] with the parameter -F 1024. Only 72 reads (coverage 5.38 %) from the sample Nigeria_MS001 and 366 reads (coverage 19.35 %) from the sample Nigeria_MS002 could be mapped to the ASFV genome.

For successful sequencing of an ASFV genome with low numbers of viral copies, we used the in-solution target enrichment strategy [13]. We selected three ASFV strains, Benin 97/1 from Africa [22], E75 from Europe [10] and Pig/HLJ/2018 from Asia [24], as reference genomes for probe design and synthesized 19 266 probes that almost completely covered the ASFV genomes of both genotypes I and II (Table S2, available in the online version of this article). We followed the protocol of iGeneTech Co., Ltd, PR China for in-solution hybrid capture sequencing and generated 1.83 and 2.21 Gb of data for Nigeria_MS001 and Nigeria_MS002, respectively. In short, genomic DNA was sheared using Scientz08-IIIC for 20 cycles of 30 s ON/30 s OFF. DNA was cleaned using Agencourt AMPure XP beads (Beckman). Library preparation was conducted using ADA and the Index kit (for Illumina) and PCR Master Mix with UDG (iGeneTech) to generate dual-indexed libraries. Hybridization of RNA baits with capture pools was performed at 50 °C for 24 h. Dynabeads MyOne Streptavidin T1 magnetic beads (Life Technologies) were used to isolate biotinylated DNA, and amplification of bead-bound enriched libraries (8–14 cycles) was performed using Post PCR Master Mix and Post PCR Primer (iGeneTech). To evaluate several mapping and assembly tools, we adopted the strategy of performing separate assemblies using the software Megahit v1.1.4 [25] and SPAdes v3.13.0 [26] and then combined the two assemblies to create the consensus sequences. The gaps in the assembled consensus sequence were filled in with ‘N’ to facilitate subsequent multiple genome sequence alignment. Finally, we determined two ASFV genome sequences of Nigeria-MS001 and Nigeria-MS002 (Table 1).

**Table 1.** Assembly information of the two Nigerian ASFV genomes

Sample ID	ASFV Nigeria_MS001	ASFV Nigeria_MS002
**Total length**	185 356 bp	182 476 bp
**Mapping rate**	99.50 %	99.88 %
**N’s**	1783 bp	106 bp
**GC (%**)	38.61 %	38.56 %
**Gaps**	865 bp	221 bp
**Indels**	33	42

### DNA alignment and phylogenetic analysis

To understand the phylogenetic relationship of the two newly generated complete genomes of ASFV from Nigeria, we downloaded 115 published ASFV genome sequences from the NCBI database (Table S1) and aligned all 117 ASFV genomes with MAFFT v7.037b [27]. A maximum-likelihood tree derived from these 117 ASFV genomes was constructed using mega Χ software incorporating the Tamura–Nei model [28].

### Molecular dating

Finally, we explored the predicted divergence date of the 27 strains in genotype I ASFV by constructing a divergence time tree using beast v2.6.7 [29]. Relaxed clock exponential and Bayesian skyline plot models were used as priors for performing 50 million heuristic search generations. The results were assessed by ESS (Effective sample size) values >200 using Tracer v1.7.2 [30].

## Results and discussion

**Fig. 1.**
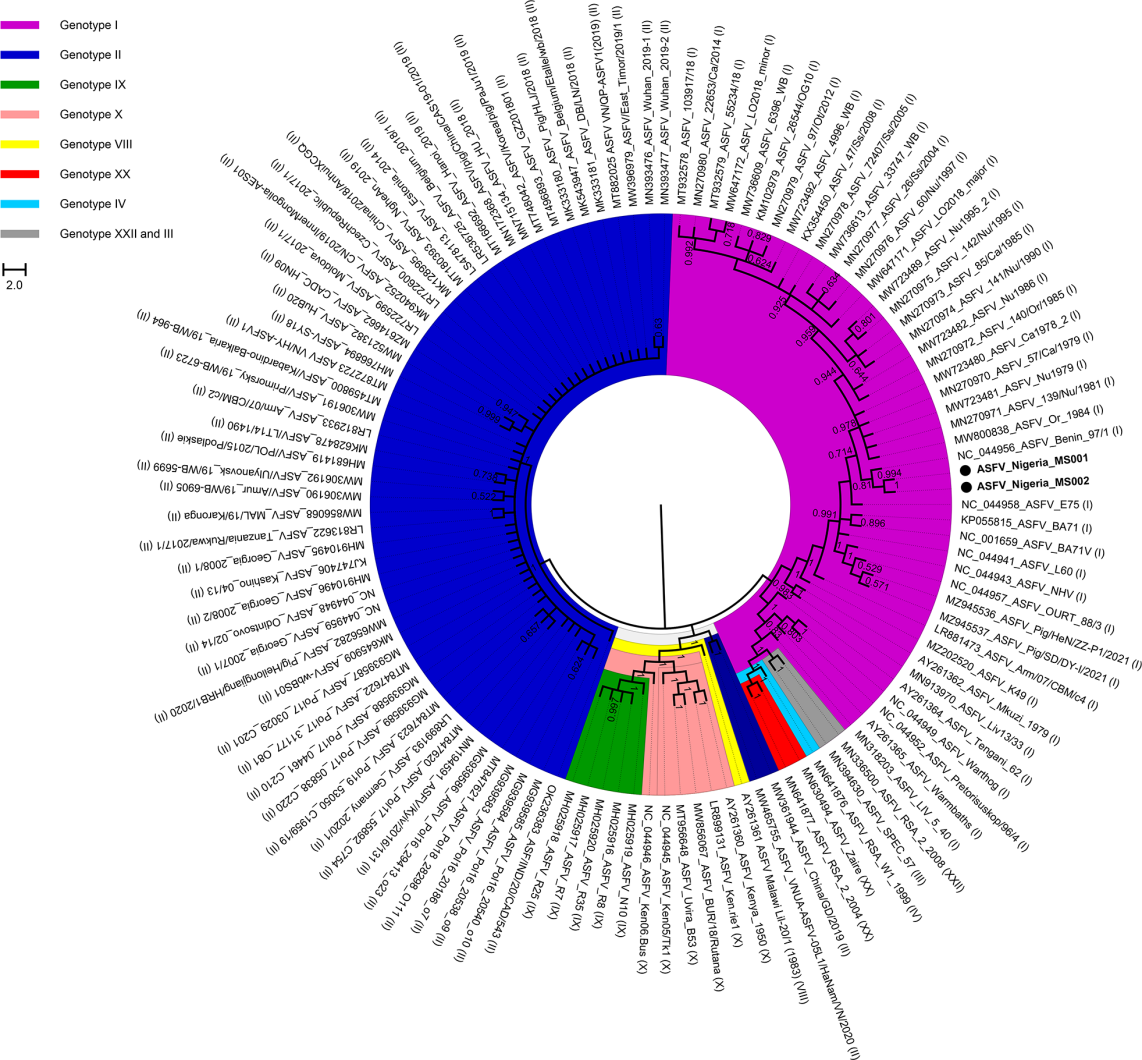
Phylogenetic tree of the two Nigerian ASFV genomes together with 115 published ASFV genomes. The phylogenetic tree was constructed using the maximum-likelihood method. Colours represent different genotypes of ASFV based on the *p72* gene.

The target capture sequencing technique provides a more cost-effective strategy to generate genomes of other organisms [13, 14]. This strategy was implemented for the first time in our study to generate ASFV genomes from the Nigerian indigenous pig population. The (near-) complete genome sequences were obtained from the two Nigerian ASFV-positive samples analysed in this study (Table 1). A phylogenetic tree based on 117 ASFV genomes revealed that the two newly assembled Nigeria-MS001 and Nigeria-MS002 genomes clustered in genotype I strain. The two genomes share 99.94 and 99.92 % genomic sequence identity with Benin 97/1 [22], respectively. Each of the two genomes has 152 ORFs (Benin 97/1 has 156 ORFs), which were exactly annotated, referring to Benin 97/1 (Fig. S1). The Nigeria-MS001 and Nigeria-MS002 genomes were in closest proximity to the Benin 97/1 strain (Fig. 1). The shared ancestry of the ASFV strains in Nigeria with the Benin 97/1 strain released in 2014 likely reflects the rapid spread of ASFV across borders in neighbouring countries in West Africa. A recent study reported the unregulated mobility of live pigs within Nigeria, which might have increased the spread of ASFV across neighbouring countries [31].

**Fig. 2.**
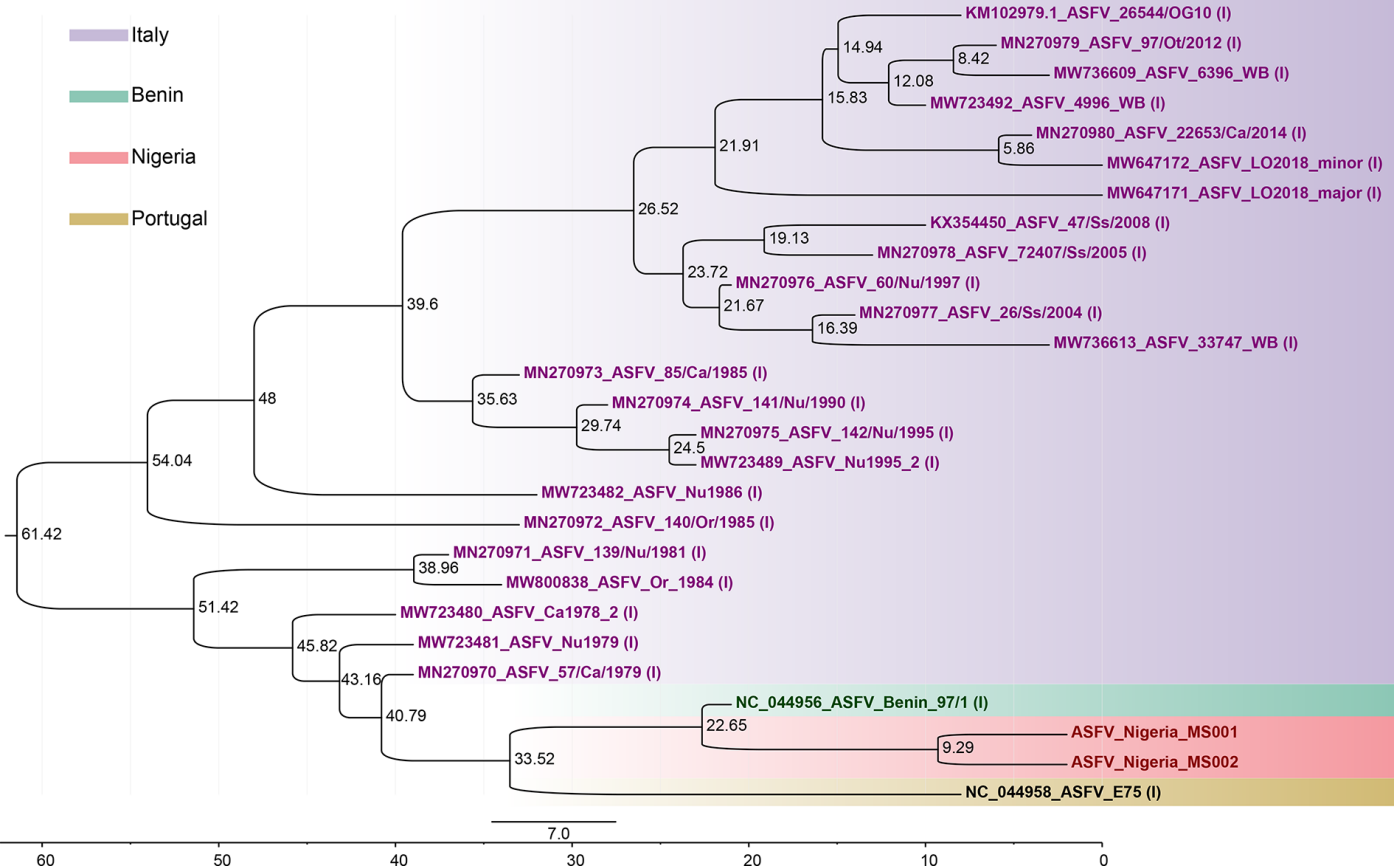
Divergence of the 27 ASFV genotype I genomes estimated using beast v2.6.7 [29]. The value at the branch node indicates the divergence time. The evolutionary rate was evaluated as 6.3×10^–6^ substitutions site^−1^ year^−1^ (95 % HPD: 4.18×10^–6^–8.45×10^–6^).

This study further revealed that all the ASFV genotype I strains from Italy formed a single lineage, which is relatively close to the West African strains, consistent with the likely origins of the Italian ASFV genotype I strains in West Africa, perhaps ultimately from the original ASFV introductions to the Iberia peninsula from Angola [32]. The Italian strains separated from the West African strains ~40.8 years ago (95 % HPD 39.0–44.7) (Fig. 2b), followed by a more recent differentiation between the strains from Benin and those in Nigeria ~22.7 years ago (95 % HPD 21.0–26.0). The divergence time trees provided more information on the differentiation and propagation of ASFV. However, further ASFV genomic data are required to confirm the exact region of introduction.

## Conclusions

This study is the first to report genomic data generated through target capture sequencing for ASFV in West Africa. The phylogeny reveals the genomic relationship of the Nigerian ASFV isolates in a global context. The target capture sequencing approach developed in this work can be applied to various biomaterials with low copy number of viruses. Given that genotype I ASFV has been isolated in PR China recently [33], retrieving genomic sequences with target capture sequencing can help trace the source and spread of ASFV.

## Supplementary Data

Supplementary material 1

Supplementary material 2

Supplementary material 3
