# Supplementary figures and images for: Target capture sequencing for the first Nigerian genotype I ASFV genome
Source: Microb Genom. 2023 Jul 25;9(7):mgen001069. doi: 10.1099/mgen.0.001069 (PMC10438811; doi:10.1099/mgen.0.001069)

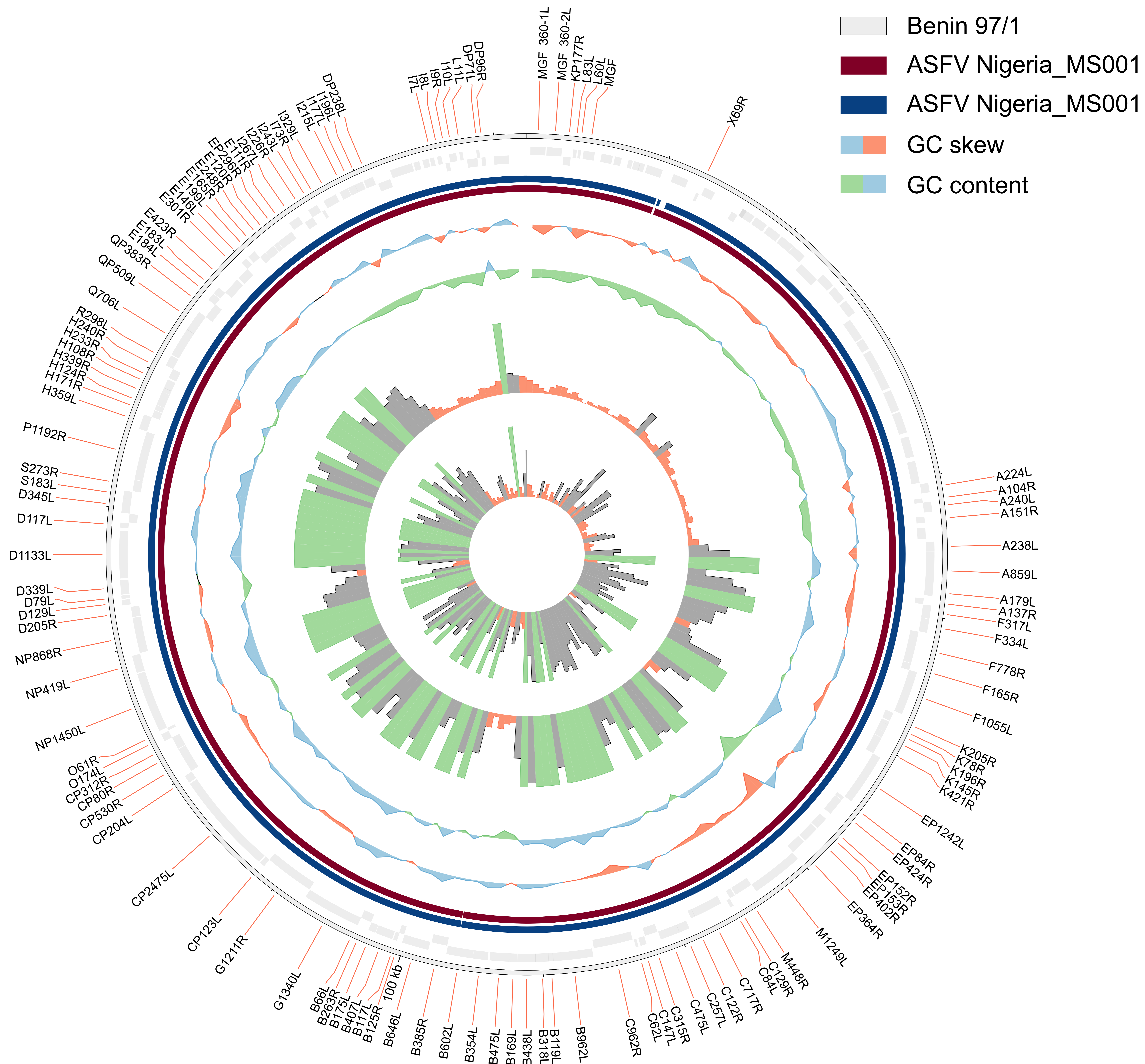

Supplement: Supplementary material 1 [file mgen-9-1069-s001.pdf]
